# Two-Channel SPR Sensor Combined Application of Polymer- and Vitreous-Clad Optic Fibers

**DOI:** 10.3390/s17122862

**Published:** 2017-12-09

**Authors:** Yong Wei, Yudong Su, Chunlan Liu, Xiangfei Nie, Zhihai Liu, Yu Zhang, Yonghui Zhang

**Affiliations:** 1 Key Laboratory of Intelligent Information Processing and Control, Chongqing Three Gorges University, Wanzhou, Chongqing 404100, China; weiyong@hrbeu.edu.cn; 2 College of Electronic ＆ Information Engineering, Chongqing Three Gorges University, Chongqing 404100, China; suyudong@hotmail.com (Y.S.); niexiangfei@hotmail.com (X.N.); 3 Chongqing Engineering Research Center of Internet of Things and Intelligent Control Technology, Chongqing Three Gorges University, Chongqing 404100, China; liuchunlan@hrbeu.edu.cn; 4 Key Lab of In-fiber Integrated Optics, Ministry Education of China, Harbin Engineering University, Harbin 150001, China; liuzhihai@hrbeu.edu.cn (Z.L.); zhangyu@hrbeu.edu.cn (Y.Z.); 5 Basic Medicine Department, Chongqing Three Gorges Medical College, Chongqing 404100, China

**Keywords:** fiber optic sensors, micro-optical devices, surface plasmon resonance, multi-channel SPR sensors

## Abstract

By combining a polymer-clad optic fiber and a vitreous-clad optic fiber, we proposed and fabricated a novel optic fiber surface plasmon resonance (SPR) sensor to conduct two-channel sensing at the same detection area. The traditional optic fiber SPR sensor has many disadvantages; for example, removing the cladding requires corrosion, operating it is dangerous, adjusting the dynamic response range is hard, and producing different resonance wavelengths in the sensing area to realize a multi-channel measurement is difficult. Therefore, in this paper, we skillfully used bare fiber grinding technology and reverse symmetry welding technology to remove the cladding in a multi-mode fiber and expose the evanescent field. On the basis of investigating the effect of the grinding angle on the dynamic range change of the SPR resonance valley wavelength and sensitivity, we combined polymer-clad fiber and vitreous-clad fiber by a smart design structure to realize at a single point a two-channel measurement fiber SPR sensor. In this paper, we obtained a beautiful spectral curve from a multi-mode fiber two-channel SPR sensor. In the detection range of the refractive rate between 1.333 RIU and 1.385 RIU, the resonance valley wavelength of channel Ⅰ shifted from 622 nm to 724 nm with a mean average sensitivity of 1961 nm/RIU and the resonance valley wavelength of channel Ⅱ shifted from 741 nm to 976 nm with a mean average sensitivity of 4519 nm/RIU.

## 1. Introduction

Surface plasmon resonance (SPR) technology is widely used for analyzing biological materials and for environment monitoring [1,2,3,4]. An SPR sensor based on Kretschmann’s structure consists of a thin layer of high-conductivity metal (usually gold or silver) covering the surface of the prism. When light reflection occurs on the surface of the prism, an evanescent field escapes into the gold film and the SRP phenomenon appears. The characteristics of the reflectance spectrum are that it is highly sensitive to the refractive index of the outside medium adjacent to the metal layer.

The basic principle of SPR sensors leads to the obtained response that sensors are affected by multiple factors. For example, in the field of biological monitoring for medications, the interaction between the sample and the probe can change the dielectric constant of a sensitive film to further affect the resonance angle or resonance wavelength (specific response). In addition, changes in components, concentrations, or temperature of a sample solution will cause an extra response. Interactions between non-target molecules in the sample solution and the sensitive film can also affect the dielectric constant of a sensitive film. In brief, the above inevitable error variances will lead to changes in the resonance angle or resonance wavelength (non-specific response). Non-specific responses will severely affect the measurement precision of an SPR sensor. Thus, it is essential to eliminate non-specific responses from the practical, measured responses. To solve this problem, a reference channel has been adopted and the specific responses are extracted by comparing different channels in order to truly realize the real-time dynamic monitoring of the interactions of the biomolecules [5,6]. In addition, in actual applications, the sensor needs to simultaneously monitor various indices such as blood glucose, cholesterol, hemoglobin, urea nitrogen, and pH in small quantities of the blood sample. Therefore, with diversification of the parameter type and the characteristics of the target sample, traditional single-channel SPR sensors cannot meet the needs of actual detection. Therefore, developing a multi-channel SPR sensor is of great urgency. Developing a novel multi-channel SPR sensor with high integration density, high throughput, and simultaneous measurement of multiple sites has become the focus of increasing concern.

Multi-channel optical fiber SPR sensors adopt mainly wavelength adjustments. Multiple parts with a sensor function are usually fabricated on the optical fiber and simultaneous monitoring of multiple sites on the same detecting structure is realized by using diverse resonance wavelengths on each sensing part. Wei Peng et al. [7] designed a multi-channel SPR fiber sensor that can detect two independent SPR signals on two sides of the same probe. Another method is to coat gold film and silver film at different positions on the same fiber and thereby manufacture two surface plasmon sensors. In theory, the discrete-type sensor structure can realize the simultaneous monitoring of two parameters and sites. However, due to the difficulty in adjusting the resonance range, a special detection channel is lacking [8]. Peng et al. [9] and Zhang et al. [10] realized the regulation of a one-channel SPR working range of distributed optical fiber SPRs by organic composite films. However, additional layers reduce the energy of interaction between the surface wave and the target medium, which leads to a decrease in sensitivity. Špačková et al. [11] and Baiad et al. [12] made use of optical grating to fabricate a multi-channel SPR sensor in a single fiber; however, a disadvantage of optical grating is its low sensitivity. Our team has fabricated a multi-channel fiber SPR sensor from a special single-mode fiber with a cone angle structure [13,14,15]. The existing problems include difficulty injecting a wide-spectrum light source into a single-mode fiber having a small fiber core, the high risk of metal films on the small fiber core being damaged by the large power intensity of the propagating light, and difficulty in spreading its use due to the high cost of the special fiber. To solve these problems, we have proposed a novel dual-channel fiber SPR sensor that combines the applications of polymer- and vitreous-clad fibers by micromachining a multi-mode fiber.

For a cascaded multi-channel SPR sensor, there is the difficulty that there is no simple and effective method to adjust the working range of the single cascade SPR resonance wavelength, and it is hard to produce two different resonance valleys to easily distinguish, at limited wavelengths, the detection range after the cascade. In our paper, we found that the resonance angle can effectively adjust the working range of the SPR resonance wavelength because with the decrease in SPR resonance angle, the working range of the resonance wavelength shows a red shift; noted after the simulation investigation on the effect of SPR incident angle on the working range of the resonance wavelength [16,17]. In the context of multiple modes coexisting in the multi-mode fiber, an investigation on the effect of the incident angle of a multi-mode fiber on SPR resonance wavelength was conducted by using a vitreous-clad optic fiber and grinding a reflection inclined plane with different angles. Based on this study, a dual-channel fiber SPR sensor—with the combined application of polymer- and vitreous-clad optic fibers—was fabricated by bare fiber grinding technology and reverse symmetry welding technology. There was a horizontal reflection plane and a grinding inclined plane, both of which were coated with a gold film of 50 nm and produced the SPR phenomenon on the polymer-clad optic fiber. Two distinguishing resonance valleys were simultaneously produced at one detection according to different SPR incident angles. To construct a dual-channel fiber SPR sensor with high performance, the following steps must be completed: reversely weld a vitreous-clad optic fiber with the same angle; plate a gold reflection film; then, collect the reflected light.

In this paper, we combined the applications of polymer- and vitreous-clad optic fibers for the first time, made use of a polymer-clad optic fiber to produce two SPR incident angles at the same measurement area, and realized the adjustment of a multi-mode fiber resonance wavelength by changing the grinding angle of the fiber in order to fabricate a dual-channel SPR sensor by wavelength-division multiplexing technology at the same measurement area. When the grinding angle was 15°, the average sensitivity of channel Ⅰ was 1961 nm/RIU and the average sensitivity of channel Ⅱ was 4519 nm/RIU, which is much higher than the average sensitivity of the traditional multi-mode fiber SPR sensor.

## 2. SPR Sensor with Two Inclined Planes on the Vitreous-Clad Optic Fiber

### 2.1. The Effect of SPR Incident Angle on the Dynamic Working Range of the Resonance Valley

**Figure 1:**
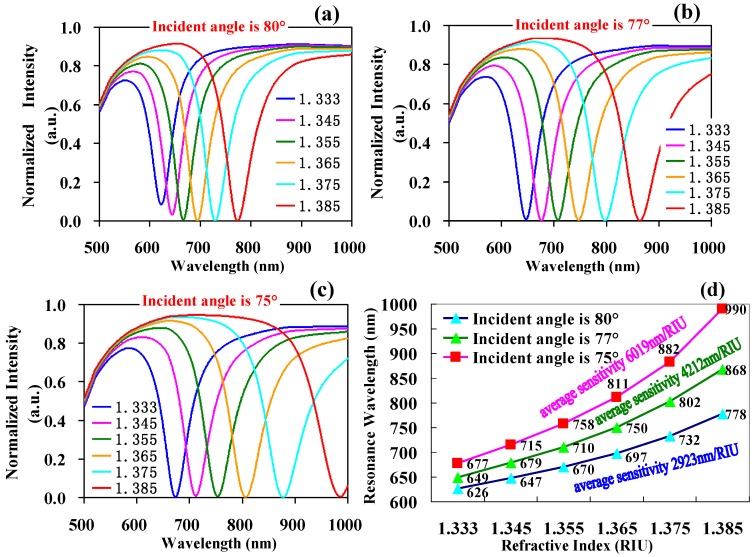
The simulation diagram of SPR signals with incident angles of (**a**) 80°, (**b**) 77°, and (**c**) 75°; (**d**) The corresponding resonance wavelength and average sensitivity of different incident angles under the detection range of the same refractive index.

When the refractive index is fixed, the dynamic working range of the SPR resonance wavelength is affected by the incident angle, the category of the metal film, the refractive index of the resonance substrate, and the refractive rate of the modulation layer. For a fiber SPR sensor, it is difficult to adjust the category of the metal film, the refractive index of the resonance substrate, and the refractive rate of the modulation layer. It is also difficult to fabricate the construct. Therefore, we fixed the metal film as a gold film, the refractive index of the resonance substrate as 1.4695, and did not add a modulation layer. The SPR reflection attenuation spectrum was respectively simulated when the incident angle was 80°, 77°, and 75° (seen in Figure 1a,b,c).

The detection range was 1.333–1.385 RIU and the dynamic working range of the resonance wavelength had a red shift from 626–778 nm to 677–990 nm (moved in the direction of a long wavelength) with a mean average sensitivity from 923 nm/RIU to 6019 nm/RIU when the incident angle of SPR decreased from 80° to 75°, respectively (seen as Figure 1d). Therefore, adjusting the SPR incident angle can effectively control the dynamic working range of the SPR resonance valley and modulate the mean average sensitivity of the sensor.

### 2.2. SPR Sensor on the Vitreous-Clad Optic Fiber by Grinding and Butt-Joint Technology

**Figure 2:**
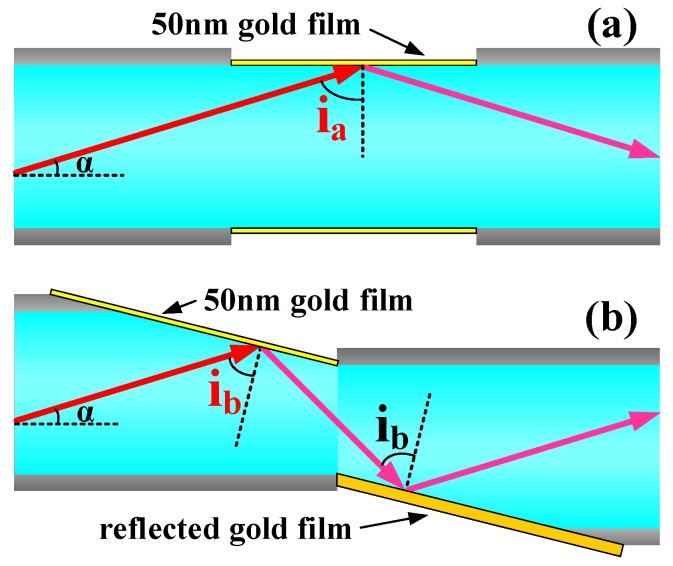
(**a**) Schematic diagram of a transmission-type SPR probe of multi-mode fiber; (**b**) Schematic diagram of a vitreous-clad fiber SPR probe with inclined structure.

The structure of the traditional multi-mode fiber transmission-type SPR probe is shown in Figure 2a. Due to the large diameter of the multi-mode fiber, the broadband light source injected into the fiber transmits in high-order mode. There are many high-order modes in the transmission of light in a multi-mode fiber and these modes mix together. When the angle α is between the mode occupied maximum power and the horizontal fiber, the angle i_a_ is between the mode occupied maximum power and the 50 nm gold film that is coated on the side face of the multi-mode fiber; namely, light will produce total reflection on the face between the gold film and the fiber core. If i_a_ (which is the incident angle of light into the gold film) meets the condition of an SPR wave vector, it will produce surface plasmon resonance and the transmission light will generate an SPR resonance valley at the resonance wavelength.

The SPR probe with an inclined structure on the vitreous-clad optic fiber is shown in Figure 2b. The structure was fabricated by butter-joint welding of two vitreous-clad multi-mode fibers with the same inclination angle of the ends. The inclined planes of the two fiber cores were respectively coated with a 50 nm gold film and a 300 nm reflection gold film. Seen in the figure, compared with the incident angle i_a_ of the traditional multi-mode fiber with transmission structure, the incident angle i_b_ between the main transmission mode of light and the gold film is smaller in the inclined structure of the vitreous-clad optic fiber. With an increase in the grinding angle of the incline plane, the incident angle was gradually reduced in order to realize the adjustment of the incident angle between the main mode of the light source and the gold film.

Through the previous simulation results in our paper, when the incident angle between the light source and the gold film was reduced gradually, the dynamic range of the SPR probe resonance wavelength produced a red shift in the sample solution with the same refractive index. Therefore, we can realize the adjustment of the dynamic range of the resonance wavelength by controlling the grinding angle of the incline plane to effectively solve the difficulty in adjusting the dynamic range of a single stage SPR in the course of building a multi-channel SPR sensor with a fiber cascade multiplexing mode. However, due to the uncertainty of the main mode angle of light transmission in the multi-mode fiber and the uncertainty of the percentage occupied by each mode in the mixture of various modes, it is hard to carry out a simulation experiment. In our paper, we have investigated, by direct experimentation, the effect of the inclined angle of the SPR probe with the inclined structure in the vitreous-clad optic fiber on the dynamic working range of SPR.

### 2.3. The Fabrication of a Probe and the Experimental Device

**Figure 3:**
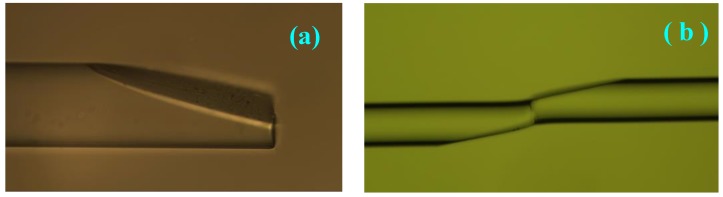
(**a**) Image of the vitreous-clad multi-mode fiber with a grinded tapered angle; (**b**) Image of a fused probe with an inclined plane structure.

The end side of a vitreous-clad multi-mode fiber (cladding diameter: 125 μm; fiber core diameter: 105 μm; numerical aperture: 0.22) was cut smooth and clamped on the fiber grinding system. The inclined structure with the incline angle *β* was fabricated by pressing the fiber near to the grinding machine and grinding the fiber at an angle of *β* between the fiber and the grinding machine. The polished multi-mode fiber inclined plane under the microscope is showed in Figure 3a where *β* = 15°. Then, the same inclined plane on the other vitreous-clad multi-mode fiber was fabricated by the same method. The end sides of the two fibers with inclined planes were clamped on the fiber welding machine and the two fibers were welded after shifting the two fibers into place (Figure 3b) by setting the welding machine upright to dislocate the two fiber cores.

We employed the plasma sputtering method to plate a 50 nm gold film on the fiber tip. The fiber probe with the structure of two inclined planes was placed on the glass slide under the microscope. The target inclined plane of the probe was adjusted to a vertically upward direction and the probe was fixed with adhesive tape. We placed the probe into the plasma cleaner (PDC-MG, Chengdu Mingheng Science ＆ Technology Co., Ltd., Chengdu, China) for 8 min to clean the ground surface of the two inclined planes of the finished probe. The probe was placed in the vacuum chamber of the plasma sputtering apparatus (ETD-2000, Beijing Elaborate Technology Development Co., Ltd., Beijing, China) to plate the fiber probe tip with a gold film. We made sure that the thickness of the gold film was 50 nm by carefully controlling the degree of vacuum, the sputtering current, and the sputtering time (the technological parameters were explored in advance). The thickness of the gold film was measured by the following method: When plating the gold film, a cover glass was placed beside the probe wih the structure of the two inclined planes. After finishing the film plating, the gold film on the cover glass was scratched in a cross shape with a backsword. The flatness of the gold film surface and the depth of the groove were observed using the three-dimensional morphology analyzer (NewView7200, Zygo, CT, USA). The depth of the groove indicated the thickness of the coated gold film. After the 50 nm gold film had been plated on one plane of the probe with two inclined planes, the probe was rotated 180 degrees under the microscope and then a 300 nm gold film was coated—as the reflecting film to collect reflected light—on the other inclined plane by using the same method.

**Figure 4:**
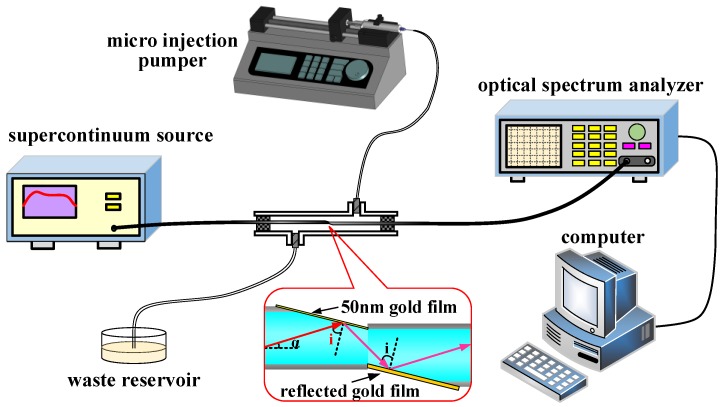
A schematic diagram of the fiber SPR sensor experiment system.

The experimental setup is shown in Figure 4. The SPR micro-sensing probe of the vitreous-clad multi-mode fiber with two inclined planes is sealed in the reaction pool. Glycerin aqueous solutions with different concentrations are prepared and the refractive index is calibrated by the Abbe refractometer (WYA-2W, Shanghai INESA, Physico optiacal Instrument Co., Ltd., Shanghai, China). The glycerin aqueous solution sample is injected into the reaction pool by a microinjection pumper (LSP01-1A, Baoding Longer Precision Pump Co., Ltd., Hebei, China) and after measurement, the waste liquid is discharged into the waste reservoir. A super continuum light source (SuperK compact, NKT Photonics, Birkerød, Denmark) is launched into the multi-mode fiber on the left of the probe. The propagating light produces total reflection on the first inclined plane and the SPR effect; the propagating light is reflected into the multi-mode fiber on the right on the second inclined plane. The light through the multi-mode fiber on the right is transmitted into the optical spectrum analyzer (AQ6373, Yokokawa, Tokyo, Japan), which collects the SPR attenuation spectrum to conduct data processing by the computer.

### 2.4. A 105 μm Vitreous-Clad Fiber Experiences Bevel Grinding and Resonance Range Changes with Incident Angle

**Figure 5:**
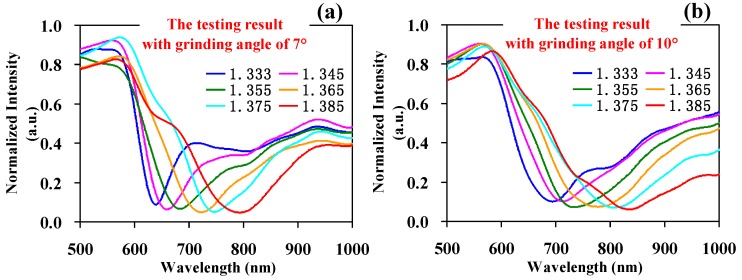

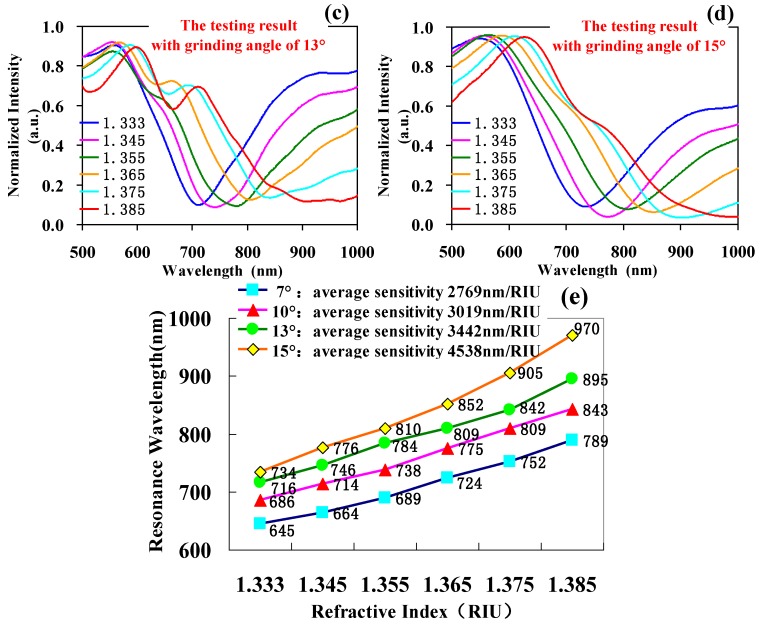
The SPR test results with grinding angles of (**a**) 7°, (**b**) 10°, (**c**) 13°, and (**d**) 15°. (**e**) The corresponding resonance wavelength and the average sensitivity of different grinding angles under the detection range of the same refractive index.

We measured glycerin aqueous solutions with refractive rates of 1.333, 1.345, 1.355, 1.365, 1.375, and 1.385 to obtain a response spectrum of solutions with different refractive indices by the SPR probe with two inclined structures (Figure 5). In Figure 5, the data was obtained by an SPR probe of vitreous-clad multi-mode fiber with two inclined planes, where the inclined grinding angles of 7°, 10°, 13°, and 15° (namely, the incident angles of the SPR basic mode as 83°, 80°, 77°, and 75°) are shown in Figure 5a,b,c,d, respectively. The resonance wavelength and mean average sensitivity of each refractive index by probes with different grinding angles are shown in Figure 5d.

According to the results of Figure 5, we can deduce the following rules:

(1) With an increase in the inclined grinding angle (7°–15°), namely a decrease in the incident angle of the SPR basic mode (83°–75°), the dynamic range of the SPR resonance valley wavelength moves in a long wavelength direction from 645–789 nm to 734–970 nm under the detection range of the same refractive index (1.333–1.385). Although there are many transmission modes in the multi-mode fiber, the existing main occupied power mode leads to a reduction in the incident angle in the multi-mode fiber with the structure of the two inclined planes, and causes a red shift of the dynamic range of the resonance valley wavelength, which has the same trend as the previous simulation results.

(2) In our previous simulation work, we found that if the basic mode is considered as the only condition, then with an increase in the grinding angle (decrease in incident angle) the mean average sensitivity of the probe is significantly augmented. Due to numerous modes in the multi-mode fiber, we found in our experiment that the average sensitivity of the probe (from 2769 nm/RIU to 4538 nm/RIU) continually increased with an increase in the inclined grinding angle from 7° to 15°.

(3) According to the experimental data, with an increase in grinding angle (the decrease in incident angle) and an augmented half-width height of the resonance valley, the degradation of the SPR resonance valley curve is more severe. The phenomenon is worse in sample solutions with a high refractive index compared to sample solutions with a low refractive index. For example, the SPR attenuation spectrum testing curve with a refractive index of 1.385—shown in Figure 5a–d (red curve in the Figure)—represents the worst curve; it has a wider and more unapparent resonance valley and a more inaccurate resonance valley wavelength value with an increase in grinding angle.

## 3. Dual-Channel SPR Sensor Combined Polymer-Clad Fiber and Vitreous-Clad Fiber

### 3.1. How to Realize a Multi-Channel of Fiber and How to Conduct Wavelength Division Multiplexing

For the fiber cascade multi-channel SPR sensor, the difficulty is that there is no easy and effective method to adjust the dynamic working range of the resonance wavelength in the single cascade SPR sensor. This leads to the difficulty in producing two distinguishable resonance valleys at the limited wavelength detection range (effective bandwidth of light source and spectrograph) after cascading the two SPR sensors. According to the investigation results of the SPR probe of a vitreous-clad multi-mode fiber with two inclined planes, with an increase in the inclined grinding angle, the dynamic range of the resonance wavelength of the probe proceeds with a red shift; this effectively realizes the adjustment of the dynamic range of the SPR resonance valley wavelength for a sample solution with the same refractive index. If the SPR probe with the structure of two inclined planes is combined with a traditional transmission multi-mode fiber at the same detection area, the dual-channel fiber SPR sensor based on wavelength-division multiplexing technology is expected to be realized.

### 3.2. A Structure Combined with Vitreous- and Polymer-Clad Optic Fibers

**Figure 6:**
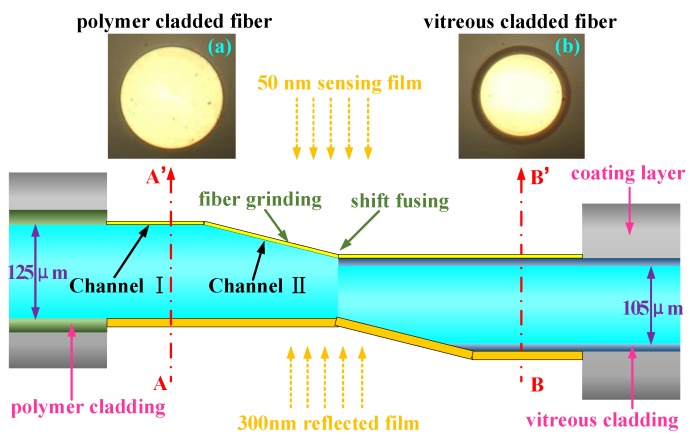
A schematic diagram of the dual-channel SPR sensing probe with the combined applications of the polymer- and vitreous-clad fibers. (**a**) Profile of the polymer-clad fiber; (**b**) profile of the vitreous-clad fiber.

To fabricate an SPR sensor on the fiber, it is necessary to discard the fiber cladding; the transmission light in the fiber core produces a total reflection on the interface of the fiber core and atmosphere to generate an evanescent field. The evanescent field interacts directly with the gold film coated on the fiber core to activate the SPR phenomenon. For the traditional multi-mode fiber SPR sensor, hydrofluoric acid is usually used to remove the fiber cladding; this causes a number of problems including difficulty in processing and an uneven surface of the fiber core after corrosion. In this paper, we made use of an SPR probe of vitreous-clad multi-mode fiber. This probe had two inclined planes that had been created by bare fiber grinding technology and reverse symmetry welding technology in order to solve the problem of the evanescent field leaking during fiber transmission. Changes in the inclined grinding angle can adjust the dynamic range of the SPR resonance wavelength. Furthermore, we combined the applications of polymer- and vitreous-clad multi-mode fibers to fabricate the SPR sensing probe with two inclined planes as shown in Figure 6.

The cladding material of the vitreous-clad fiber was quartz cladding, which cannot be directly wiped out. The cladding material of the polymer-clad fiber was a hard polymer coating, which can be wiped out by a blade machine. In this paper, we adopted a polymer-clad quartz fiber with a 125 μm diameter fiber core (PTIF125/140/250-HT37, NewPion Photonics, Jiangsu, China) and mechanically removed the polymer cladding to achieve under the microscope an end face as shown in Figure 6a. The horizontal part of the bare fiber core of the polymer-clad fiber, as the traditional transmission-type (inclined angle of fiber is 0°), was the first channel of the SPR probe and the inclined plane after grinding the end face was the second channel of the SPR probe (shown in Figure 6). We adopted the vitreous-clad multi-mode fiber (PTIU105/125/250-22, NewPion Photonics, Jiangsu, China) and mechanically removed the cladding to achieve an end face under the microscope as shown in Figure 6b. The same inclined plane was obtained after grinding and the light was collected by reversal core-shift welding technology. After stripping the cladding of the polymer-clad fiber, the area of the bare fiber core formed the horizontal sensing area Ⅰ and the grinding end tip formed the inclined sensing area Ⅱ. The two areas were coated with 50 nm sensing films and the grinding inclined plane of the vitreous-clad fiber was coated with 300 nm reflection gold film. Compared with the fiber cascade dual-channel SPR sensor based on wavelength division multiplexing technology, we fabricated the dual-channel SPR sensor with an advantage of detecting in the same sample area with the same temperature and the same non-specific response of refractive index for the two channels; this was to truly realize the compensation of temperature and non-specific responses.

### 3.3. Results

**Figure 7:**
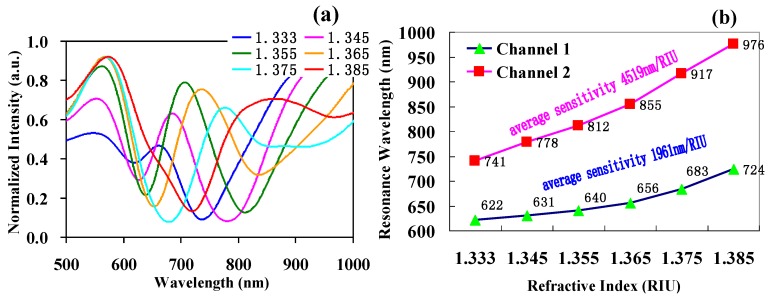
The test results of a dual-channel SPR sensor. (**a**) The test spectrum of the refractive index; (**b**) comparison of the dual-channel resonance wavelength and average sensitivity.

Using the experimental testing system shown in Figure 4, we conducted a dual-channel experiment of the SPR sensing probe having two inclined planes and the combined applications of polymer- and vitreous-clad fibers. The test result is shown in Figure 7. In Figure 7a, solutions under different refractive indices have the respective spectrum curve. Every spectrum curve produces two distinguishable SPR resonance valleys including the resonance valley under short wavelength produced by the horizontal SPR sensing area Ⅰ and the resonance valley under long wavelength produced by the inclined SPR sensing area Ⅱ. When the refractive index of the solution changes from 1.333 to 1.385, the resonance wavelength under every sensing area moves in a long wavelength direction with an increase in the refractive index of the sample solution; the two resonance valleys under the two sensing areas move together. Shown in Figure 7b, the resonance wavelength of channel Ⅰ moves from 622 nm to 724 nm with a mean average sensitivity of 1961 nm/RIU; the resonance wavelength of channel Ⅱ moves from 741 nm to 976 nm with a mean average sensitivity of 4519 nm/RIU.

## 4. Conclusions

In this paper, we made use of a series of features; for example, the organic polymer cladding of the polymer-clad fiber was mechanically stripped together with the coating layer; only the coating layer was mechanically stripped for the vitreous-clad fiber, with the silicon dioxide cladding and fiber core remaining. After a smart design of the structure, we fabricated a novel dual-channel SPR sensor by the combined application of a polymer-clad fiber and a vitreous-clad fiber by bare fiber grinding technology and shift welding technology. The novel SPR sensor can be used to simultaneously detect multiple analytes in mixtures; a reference channel is adopted to compensate for temperature and eliminate non-specific interference.

By bare fiber grinding technology, the end-tip cladding of the fiber is stripped and the fiber core is exposed to the atmosphere. When light is transmitted in the fiber core, an evanescent field is produced on the interface between the grinded area and the atmosphere; this fabricates the SPR sensor after coating with a 50 nm gold film. This method avoids a number of disadvantages that occur during fabrication of the traditional multi-mode fiber SPR sensor. For the traditional multi-mode fiber SPR sensor, hydrofluoric acid is usually used to remove the fiber cladding but it causes a number of problems including dangers associated with processing and the uneven surface of the fiber core after corrosion; in addition, the gold film coating has a great effect on the properties of the SPR sensor, and it is hard to fabricate the probe with consistent performance. The structure that we have proposed can realize the change in the SPR incident angle by altering the grinding angle and thereby adjust the dynamic working range of the SPR resonance wavelength. This solves the difficulty in producing a sensing area with different resonance wavelengths for the fiber SPR sensor. It further solves the problems of multi-channel measurements. On the basis of investigating the effect of the grinding angle on dynamic range changes of the SPR resonance valley wavelength and sensitivity, we combined polymer-clad fiber and vitreous-clad fiber by a smart design structure to realize a two-channel measurement fiber SPR sensor at a single point. In this paper, we obtained experimental results with a beautiful spectral curve from a multi-mode fiber two-channel SPR sensor. The detection range of the refractive rate was between 1.333 RIU and 1.385 RIU. For channel Ⅰ the mean average sensitivity was 1961 nm/RIU and for channel Ⅱ the mean average sensitivity was 4519 nm/RIU.
